# Exercise-specific post-translational modification signatures: unveiling precise regulatory mechanisms of molecular exercise language and cellular adaptation

**DOI:** 10.3389/fspor.2026.1765170

**Published:** 2026-02-13

**Authors:** Yinghao Shen, Zhujun Mao, Heming Chen, Wenyue Zhu, Qianhui Guan, Yupeng Yang, Junjie Liu, Li Li

**Affiliations:** 1 Graduate School, Harbin Sport University, Harbin, China; 2 School of Basic Medicine, China Three Gorges University, Yichang, China; 3 College of Science and Technology, China Three Gorges University, Yichang, China; 4 School of Exercise Science and Health, Harbin Sport University, Harbin, China

**Keywords:** exercise-specific PTM signatures, inter-organ communication, machine learning, metabolic memory, post-translational modifications, precision exercise prescription

## Abstract

Exercise reshapes cellular function and intercellular communication through dynamic post-translational modifications (PTMs) that fine-tune protein activity and inter-organ signaling. However, the traditional aerobic–anaerobic dichotomy does not fully capture PTM-driven regulatory logic across exercise modes. This review centers on the muscle–brain and muscle–liver axes and proposes an operational concept of exercise-specific PTM signatures, emphasizing acetylation, ubiquitination, and lactylation as core PTMs implicated in metabolic memory and adaptive remodeling. To connect exercise intensity with metabolic improvement, we introduce the PTM threshold theory and outline how integrated exercise–PTM–disease target databases, coupled with machine-learning approaches, can support personalized exercise prescription and translation toward exercise pharmacology and rare-disease rehabilitation. Overall, PTM-centered regulatory networks provide a unifying and actionable framework for decoding exercise adaptation and prioritizing therapeutic strategies.

## 1 Introduction

PTMs are key molecular mechanisms that finely regulate cellular signaling cascades and functional outputs while enabling dynamic cellular adaptation to physiological and environmental changes (1, 2). Exercise is typically categorized into aerobic and anaerobic types based on energy systems and metabolic traits (3, 4), a framework that serves well for general exercise guidance and basic physiological understanding but overlooks the complex molecular regulatory differences triggered by distinct exercises at the cellular level (5). Exercise-specific PTM signatures refer to distinct PTM patterns induced by different types of exercise. Current studies suggest that these patterns might be unique to each exercise modality. However, direct evidence in exercise science remains limited, and further research is needed to validate this concept (6, 7). A full understanding of these exercise-specific PTM signals is essential for developing precise exercise plans and targeted interventions for disease prevention and rehabilitation (8).

Aerobic and anaerobic exercises differ fundamentally in energy metabolism and physical demands: aerobic exercise primarily relies on oxidative phosphorylation to enhance fatty acid metabolism and promote mitochondrial biogenesis (4, 9), while anaerobic exercise induces rapid ATP turnover and lactate accumulation (10). These metabolic differences alter cellular homeostasis, protein function, and gene expression by modulating the activation of signaling cascades and molecular pathways. For instance, aerobic exercise boosts total antioxidant capacity and superoxide dismutase (SOD) activity, demonstrating stronger enhancement of the body's antioxidant defenses compared to anaerobic exercise (9). In contrast, anaerobic exercise elevates purine turnover and glycolytic flux, reflecting its unique metabolic stress and recovery processes (4, 11, 12). These metabolic and signaling disparities highlight gaps in traditional exercise classification, which fails to account for the unique molecular mechanisms underlying cellular responses to exercise.

Key PTMs including phosphorylation, acetylation, ubiquitination, glycosylation, and methylation play critical roles in regulating molecular-level exercise adaptation. These reversible mechanisms regulate protein activity, localization, stability, and interactions to orchestrate complex signaling networks (13–15), governing skeletal muscle contraction, energy utilization, and structural remodeling in response to different exercises (16). For example, high-intensity aerobic interval training predominantly promotes mitochondrial protein acetylation, emphasizing post-translational regulation of energy-producing machinery. Resistance exercise induces phosphoproteomic changes in contractile and cytoskeletal proteins that align with strength gains; and acute aerobic exercise triggers more pronounced phosphoproteomic and metabolomic responses than resistance exercise, indicating exercise type-dependent signaling patterns (17–19). These findings confirm that PTMs act as a finely tuned molecular language translating distinct exercise types into specific cellular adaptations.

Exercise does not induce isolated PTMs but rather complex combinations and crosstalk between distinct PTM types, forming unique patterns that are vital for the molecular regulation of exercise adaptation. They shape metabolic control, interorgan communication, and cellular reprogramming. Phosphorylation-acetylation crosstalk modulates key metabolic enzymes and transcription factors to influence glucose and lipid metabolism (17, 20). Ubiquitination and ubiquitin-like modifications regulate protein turnover and signaling pathways, critical for maintaining muscle protein homeostasis during exercise adaptation (21). Such PTM crosstalk enhances the specificity and adaptability of molecular responses, enabling precise regulation of cellular functions based on exercise types, intensities, and durations (22, 23). Recent studies have also documented tissue specificity and sexual dimorphism in exercise-induced PTMs, highlighting the complexity of whole-body adaptation (24, 25).

“Exercise-specific PTMs” denote the concept that distinct exercises induce unique PTM patterns acting as molecular codes guiding cellular adaptation, a notion highly relevant to precision medicine and exercise science (26, 27). By integrating multi-omics data including transcriptomics, proteomics, and metabolomics, researchers can identify PTM biomarkers (24, 28) that predict individual exercise responses and inform personalized exercise plans. PTMs underpin numerous exercise-related benefits such as improved metabolic regulation, cardiovascular health, immune function, and neuroplasticity, and understanding their roles can guide targeted interventions for chronic conditions like atherosclerosis, diabetes, and neurodegeneration (29–31). For example, aerobic and resistance exercise exert distinct effects on PTM-mediated modulation of inflammatory pathways such as the NLRP3 inflammasome, influencing systemic inflammation and disease risk (32). In addition, PTMs regulate muscle protein synthesis and breakdown to promote exercise-induced muscle growth and functional improvement (33, 34).

This review synthesizes current knowledge on exercise-specific PTMs, emphasizing their role as a distinct molecular language governing cellular adaptation to different exercises. It first addresses the limitations of traditional aerobic-anaerobic exercise classification, focusing on its failure to capture molecular regulatory diversity. Next, it explores the crosstalk between distinct PTMs in mediating exercise adaptation, highlighting their combined functions and tissue-specific effects. The review introduces the concept of “exercise-specific PTM signatures” and discusses their potential as biomarkers for targeted exercise prescription and disease intervention. Finally, it covers emerging technologies and analytical methods for PTM identification and functional characterization, emphasizing the importance of this molecular framework for translational research. This work seeks to advance understanding of exercise biology by delineating the complex molecular interactions underlying exercise-induced PTMs, in turn facilitating the development of customized exercise programs for health enhancement and disease management.

## 2 Definition and classification of exercise-specific PTM signatures

### 2.1 Limitations of traditional exercise classification and advances from the PTM perspective

**Figure 1:**
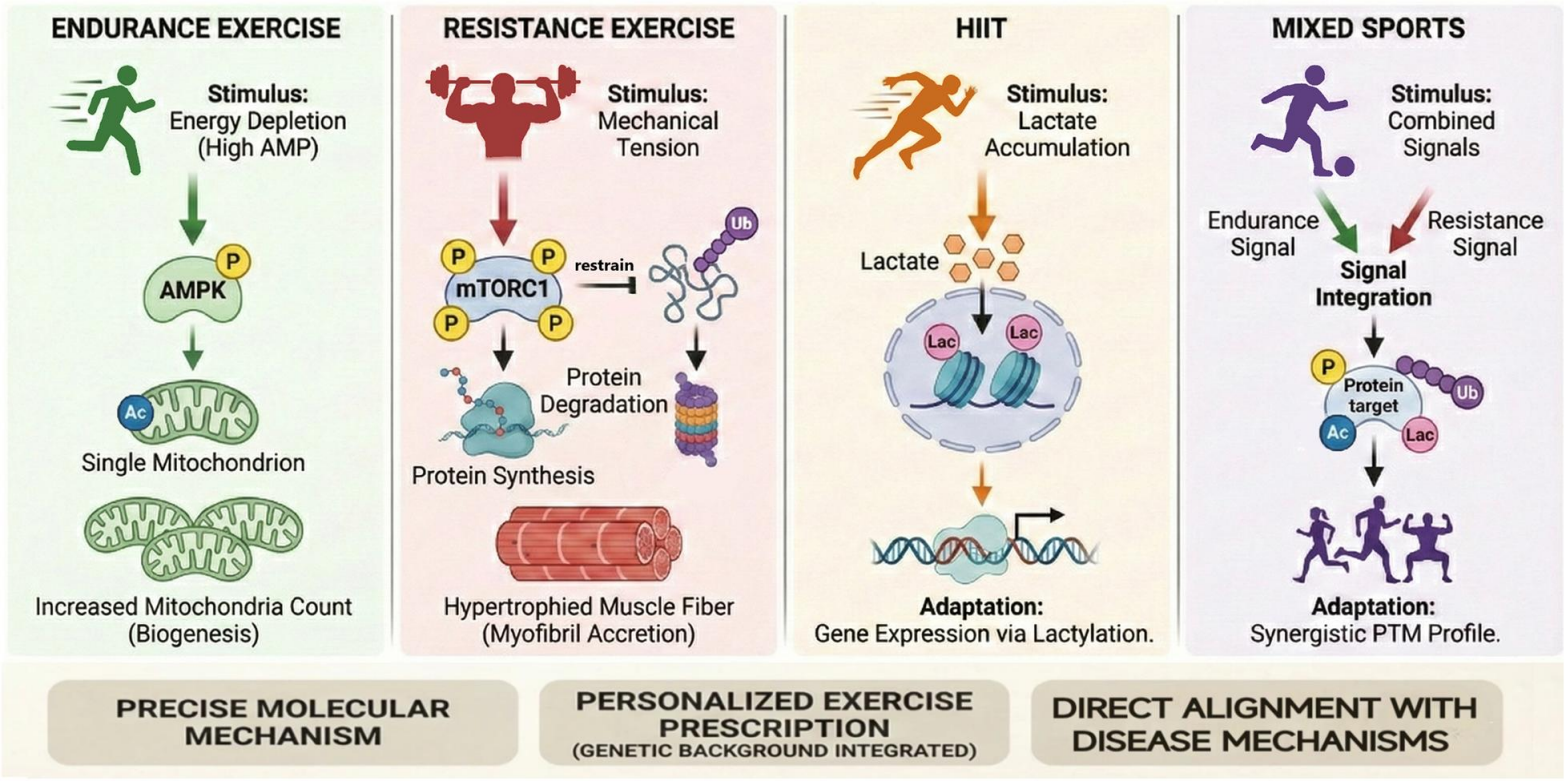
A molecular framework for exercise classification based on specific PTM signatures. Figure 1 describes a molecular framework for exercise classification based on specific PTM signatures. Unlike the conventional metabolic classification, this framework aligns exercise modes with distinct Post-Translational Modification (PTM) fingerprints. Endurance exercise activates the AMPK-mitochondrial axis, involving mitochondrial acetylation (Ac) and phosphorylation; Resistance exercise stimulates mTORC1-mediated protein synthesis via phosphorylation while limiting degradation through the modulation of ubiquitination (Ub); HIIT promotes adaptation via lactate accumulation and histone lactylation (Lac); and Mixed sports integrate these pathways into a synergistic PTM profile. This precise mapping provides a molecular basis for personalized exercise prescription. P, Phosphorylation; Ub, Ubiquitination; Lac, Lactylation; Ac, Acetylation.

Conventional exercise classification typically divides physical activity into aerobic and anaerobic types, based primarily on energy metabolism pathways and physiological responses (35, 36). This binary classification overlooks the subtle molecular variations induced by distinct exercise modes at the cellular level (37) (Figure 1). Aerobic exercise relies more on oxidative phosphorylation and mainly improves endurance. Anaerobic exercise relies more on glycolysis and supports rapid, high-force output. Endurance training increases mitochondrial content and oxidative capacity, often involving AMPK and PGC-1α signaling (38, 39). It also increases muscle capillary density through angiogenic signals such as VEGF and nitric oxide, which improves oxygen delivery and fatigue resistance (40). By contrast, resistance-style training activates mechanosensitive mTORC1 signaling and supports ribosome biogenesis, which increases muscle protein synthesis and drives hypertrophy (41, 42). Early strength gains are also explained by neural changes, such as improved motor unit recruitment and firing behavior (43). However, these metabolic distinctions fail to fully capture the complexity of intracellular signaling and molecular changes triggered by diverse exercises.

PTMs regulate protein function via reversible chemical modifications including phosphorylation, acetylation, ubiquitination, lactylation, glycosylation, and methylation, providing a precise framework for understanding cellular adaptation to exercise (34, 44). PTMs play a pivotal role in the molecular regulation of exercise adaptation, acting as rapid, reversible molecular switches that connect external stimuli to intracellular responses and enable real-time cellular adaptation to specific exercise triggers. Exercise-specific PTM signatures transcend traditional classification approaches, conferring each exercise type a unique molecular fingerprint that reflects the exercise's impact on the body's regulatory patterns (8, 12, 45).

This approach enables more precise classification of exercise types through their unique PTM profiles, enhancing understanding of the molecular mechanisms underlying exercise-induced effects on cellular function and adaptation (46, 47). PTM profiling allows researchers to investigate how distinct exercises impact individuals, moving beyond traditional metabolic classifications to uncover specific signaling pathways and adaptive mechanisms previously obscured by conventional frameworks. The innovation of the PTM perspective lies in its capacity to capture the dynamic, context-specific characteristics of exercise-induced molecular regulation, providing a new framework for exercise biology and supporting personalized training strategies (48, 49).

### 2.2 PTM combination patterns induced by different exercise types

Different exercise types induce unique PTM combinations that likely drive specific cellular changes. These patterns are suggested by current research, though further studies are required to directly confirm how they contribute to exercise adaptation. Endurance running strongly activates the AMP-activated protein kinase (AMPK) pathway, where AMPK functions as a metabolic sensor that enhances mitochondrial biogenesis and energy homeostasis (50, 51). AMPK activation increases protein acetylation particularly on histones and metabolic enzymes, which modulates gene expression and enzyme activity to sustain long-term oxidative metabolism (52). Acetylation also regulates the mechanistic target of rapamycin (mTOR) pathway, a key regulator of protein synthesis and cell growth that balances anabolic and catabolic processes during prolonged endurance running (53–55). Strength training primarily relies on the Fn14-TRAF6 signaling pathway, promoting muscle hypertrophy and structural remodeling through ubiquitination. Ubiquitination serves as a quality control mechanism that degrades damaged or unnecessary proteins, facilitating the reconstruction of muscle contractile structures. High-intensity interval training (HIIT), characterized by repeated intense efforts, is associated with elevated lactylation levels, a lactate-derived PTM from lactate metabolism (56–58). Lactylation modifies histones and other proteins and may influence gene programs related to metabolic stress and recovery. Histone lysine lactylation adds a lactyl group to lysine residues on histone tails (H3K18la). This mark is linked to more open chromatin and higher transcription of target genes (59–61).

Ball sports require complex physical capabilities involving rapid direction changes, technical movements, and intermittent bursts of activity, integrating a well-coordinated network of diverse training-related elements (56, 57). Here, ball sports are presented only as one example of mixed-demand (intermittent) exercise rather than a single standardized training model. Accordingly, physical demands vary across sports and even across playing positions. For example, in American football, player-tracking data show clear position-specific profiles, with skill positions accumulating more high-speed running and accelerations, whereas linemen typically show the lowest running loads and greater collision/contact exposure (62). Similarly, in basketball, guards generally perform more distance, accelerations, and change-of-direction actions, while centers are exposed to more contact, rebounds, and jumping actions (63, 64). These sport- and position-specific load profiles likely shift the balance of mechanical, metabolic, and inflammatory signals and may therefore lead to different PTM signatures (5, 17). Phosphorylation, acetylation, ubiquitination, and lactylation coordinate here to regulate neuromuscular coordination, energy metabolism, and inflammation (65). The crosstalk of these PTMs in ball sports reflects complex molecular regulation that adapts to the sport's physical and biomechanical demands (Table 1). Accordingly, future PTM-omics work in ball sports should, where possible, stratify participants by sport and position/role (and report exposure) to avoid averaging out position-specific signatures (68). Understanding these PTM patterns clarifies the molecular basis of exercise specificity and identifies targets to optimize training and recovery strategies tailored to the unique needs of each exercise type (23, 69, 70).

**Table 1:** PTM effects of different exercise modalities.

Exercise modality	Core PTM types	Main physiological effects	References
Endurance Exercise (Long-distance Running)	Acetylation, Phosphorylation	Enhanced mitochondrial biogenesis, improved oxidative metabolism	(13, 17)
Resistance Training (Strength Training)	Phosphorylation, Ubiquitination	Activated muscle protein synthesis, muscle hypertrophy	(41, 66)
High-Intensity Interval Training (HIIT)	Lactylation, Acetylation	Metabolic stress adaptation, glycolipid metabolic reprogramming	(58, 67)
Ball Sports (Complex Modality)	Phosphorylation + Ubiquitination + Lactylation (synergistic)	Neuromuscular coordination, improved multi-dimensional exercise performance	(62, 65)

This table shows key exercise-induced PTMs and their physiological pay-offs. Core, reversible marks (acetylation, phosphorylation, etc.) gate signalling pathways unique to each modality; downstream effects improve performance and metabolic control. In complex sports, overlapping PTMs jointly tune neuromuscular coordination, energy supply and inflammation to meet mixed demands.

### 2.3 The role of exercise-specific PTM signatures in inter-organ communication

**Figure 2:**
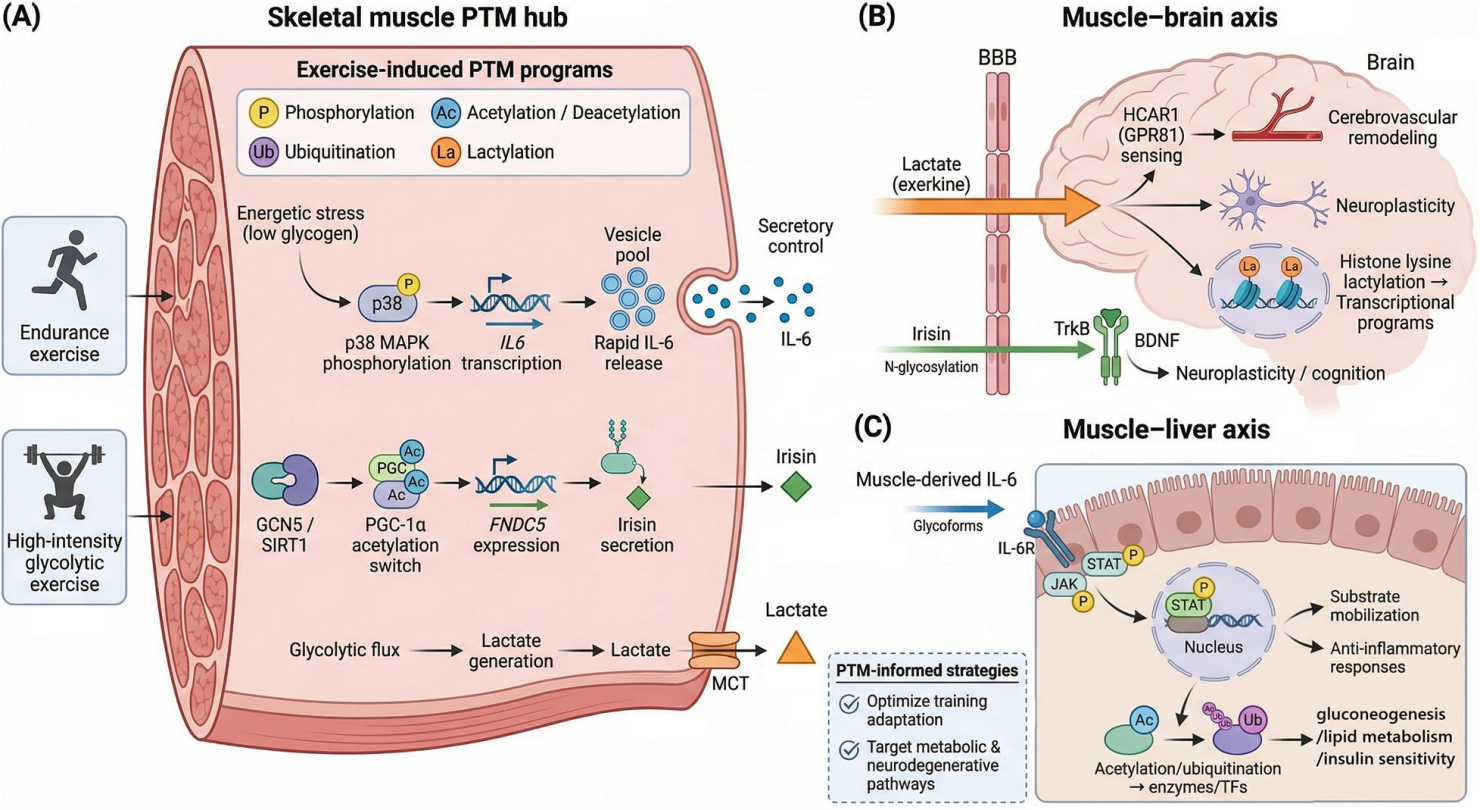
Exercise-induced systemic inter-organ communication facilitated by PTM signatures. Figure 2 depicts how exercise-induced post-translational modifications (PTMs) in skeletal muscle act as a central PTM signaling hub. They coordinate systemic inter-organ crosstalk. **(A)** Key PTMs include phosphorylation (p38 MAPK), acetylation/deacetylation (GCN5/SIRT1–PGC-1α), lactylation (histone lysine lactylation), and ubiquitination. These PTM programs tune secretory control of major exercise factors, including IL-6, irisin (FNDC5), and lactate (export via MCT). **(B)** The muscle–brain axis is mediated by lactate as an exerkine that crosses the BBB and signals via HCAR1 (GPR81) to support cerebrovascular remodeling and neuroplasticity, while irisin (stabilized by N-glycosylation) engages BDNF/TrkB signaling to promote neuroplasticity and cognition; lactate-linked histone lysine lactylation is shown as a transcriptional route. **(C)** The muscle–liver axis is driven by muscle-derived IL-6 (including glycoforms) activating hepatic IL-6R and JAK–STAT phosphorylation, together with PTM-regulated metabolic enzymes/transcription factors to coordinate substrate mobilization, anti-inflammatory signaling, gluconeogenesis, lipid metabolism, and insulin sensitivity. The bottom panel highlights translational applications, emphasizing PTM-informed strategies for training optimization and disease-relevant pathways.

Exercise-induced PTM signatures extend beyond local tissue adaptations and help coordinate systemic inter-organ communication that supports whole-body homeostasis. The muscle–brain axis illustrates this cross-talk, where PTMs contribute to neuroplasticity and cognitive function. In muscle cells, acetylation and phosphorylation can tune the production and release of factors such as FNDC5 (irisin) and IL-6 during exercise (71–73) (Figure 2). p38 MAPK phosphorylation links energetic stress (low glycogen) to IL6 transcription (74). A pre-formed intramyofiber vesicle pool can enable rapid IL-6 release during contractions (75). PGC-1α acetylation–deacetylation (GCN5/SIRT1) acts as a PTM switch that modulates FNDC5 expression and irisin output with training (76). During high-intensity glycolytic exercise, lactate rises and functions as a metabolite-derived exerkine that supports brain function and neuroplasticity (77, 78). Lactate can cross the blood–brain barrier and promote hippocampal BDNF/TrkB signaling, and lactate sensing via HCAR1 has been linked to exercise-induced cerebrovascular remodeling (79, 80). Lactate can also fuel histone lysine lactylation, which can stimulate transcriptional programs and link glycolytic bouts to downstream gene regulation (59). Secreted proteins can be modified, with N-glycosylation supporting FNDC5/irisin stability and secretion and IL-6 glycoforms modulating signaling duration and clearance (81).

During prolonged aerobic exercise, skeletal muscle is a major source of circulating IL-6 (82, 83). IL-6 receptor signaling engages JAK–STAT phosphorylation and helps coordinate substrate mobilization and anti-inflammatory responses across tissues (83). PTM-driven metabolic pathways shape the muscle–liver axis and regulate nutrient utilization (84–87). Acetylation and ubiquitination reprogram key enzymes and transcription factors in muscle and liver, enhancing gluconeogenesis, lipid metabolism, and insulin sensitivity to support performance and recovery (88). PTMs act as hubs in cross-organ signaling networks that integrate endocrine, immune, and metabolic cues (89, 90). Exercise-induced changes in PTM activity modulate the release of hormones, cytokines and metabolites. These molecules influence systemic inflammation, oxidative stress and tissue repair (91–93). These mechanistic links support PTM-informed strategies to optimize training adaptation and to target metabolic and neurodegenerative disease pathways (93–95).

## 3 PTMs of proteins induced by exercise: regulatory mechanisms at multiple levels and tailored metabolic adaptation

### 3.1 Lactylation, acetylation, and ubiquitination in a three-dimensional regulatory network

Lactylation, acetylation, and ubiquitination collaboratively form a complex three-dimensional regulatory network that governs metabolic alterations, particularly in relation to exercise-induced metabolic memory. Lactylation is a recently identified PTM involving the addition of lactyl groups to lysine residues, which promotes histone modifications and enhances the expression of genes related to fatty acid oxidation. Although evidence supports the role of lactylation in various cell types, most studies have focused on macrophages, cancer biology, or *in vitro* models. The exercise-specific and tissue-specific roles of lactylation in muscle and brain remain largely unexplored. High lactate levels, indicative of increased glycolytic flux during intense exercise, are thought to lead to histone lactylation, which relaxes chromatin and activates metabolic gene transcription. However, further research is needed to validate this mechanism specifically within muscle and brain tissues during exercise. This accelerates fatty acid degradation and enhances energy availability (96, 97). This epigenetic mechanism links metabolic alterations to gene expression modifications, facilitating rapid cellular adaptation to varying energy requirements.

Acetylation is a significant PTM that regulates mitochondrial dynamics and energy metabolism by altering the function of mitochondrial proteins and transcription factors. The acetylation status of mitochondrial enzymes modulates their activity, refining oxidative phosphorylation and ATP synthesis (98–100). Furthermore, the acetylation of histones and non-histone proteins influences chromatin accessibility and the transcriptional programs governing mitochondrial biogenesis and function, which are crucial for maintaining energy homeostasis during and after exercise (101, 102). This change is therefore a key link between the cellular energy state and gene regulatory networks.

**Figure 3:**
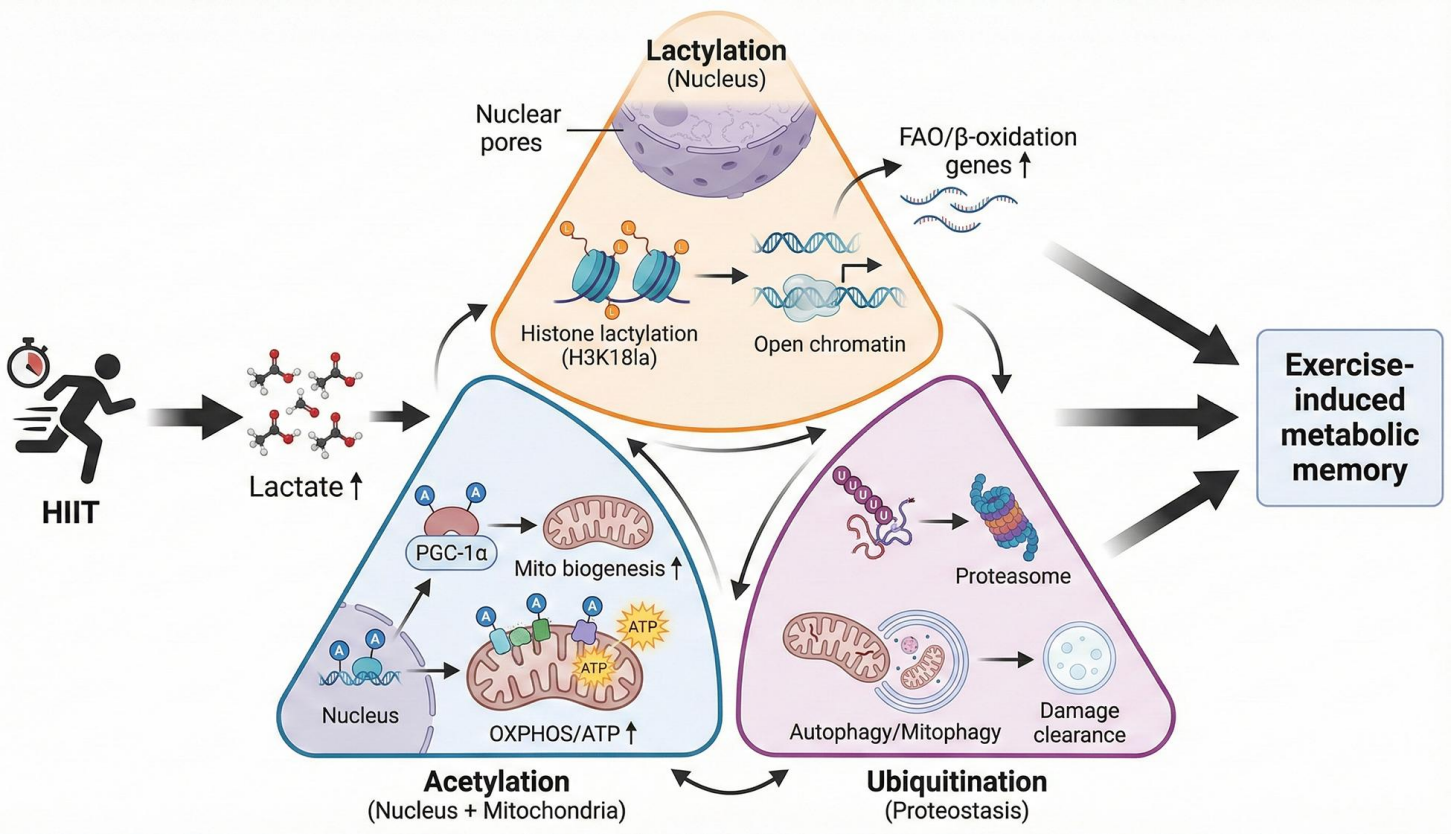
Coordinated regulation of lactylation, acetylation, and ubiquitination in the establishment of exercise-induced metabolic memory. Figure 3 depicts the process by which during high-intensity exercise, surging lactate drives histone lactylation, opening chromatin for *β*-oxidation genes. Parallel acetylation boosts mitochondrial enzymes and nuclear PGC-1*α*, enhancing ATP output and biogenesis. Ubiquitination then clears deacetylases, damaged myofibrillar proteins and faulty mitochondria, sustaining high acetylation and autophagic flux. The three PTMs operate as a self-reinforcing network where lactylation launches transcription, acetylation expands metabolic capacity, and ubiquitination preserves proteostasis, collectively establishing metabolic memory in muscle.

Ubiquitination regulates protein quality by tagging damaged or unnecessary proteins for proteasomal degradation and modulating autophagy processes. Cells regulate proteostasis and eliminate damaged mitochondrial components via selective ubiquitination, a process crucial for cellular adaptation to metabolic stress. Ubiquitination alters the signaling pathways governing metabolism and inflammation, thereby influencing cellular responses to exercise-induced stress (97, 103). The balance between ubiquitination and deubiquitination ensures the proper turnover of vital metabolic regulators and maintains cellular homeostasis. These PTMs constitute a network of regulatory pathways, lactylation facilitates the function of genes involved in fatty acid degradation, acetylation alters mitochondrial activity and energy utilization, while ubiquitination regulates protein degradation and cellular recycling processes. Their coordinated actions allow precise control of metabolic pathways and cellular adaptation to exercise stimuli, laying the molecular groundwork for metabolic memory (Figure 3). Understanding this three-dimensional PTM network offers insights into the mechanisms through which exercise promotes lasting metabolic improvements and may inform therapeutic strategies for metabolic disorders (104, 105).

### 3.2 The “PTM threshold” hypothesis and Its molecular basis

The “PTM threshold” hypothesis proposes that a certain exercise intensity is necessary to induce significant PTM modifications that could trigger lasting metabolic improvements. However, it remains a hypothesis due to the lack of direct experimental evidence showing a discrete “threshold” for PTM modifications. Current research suggests that the relationship between exercise intensity and PTM modification is more likely continuous, with dose-response relationships where incremental changes in PTM accumulation lead to gradual metabolic adaptations, rather than a clear-cut threshold (106–108). Thus, further experimental and longitudinal studies are needed to determine if a specific PTM threshold exists and to clarify whether dose-dependent or threshold-like behavior governs metabolic memory (109–111). The molecular threshold is dynamically regulated through the interactions of PTM writers, erasers and readers, which alter the quantity and duration of modifications. Acetyltransferases and deacetylases regulate acetylation levels, while ubiquitin ligases and deubiquitinases modulate the extent of ubiquitination. Acetyltransferase-like enzymes such as p300 catalyze lactylation, and their activity is influenced by intracellular lactate levels that reflect metabolic flux (96, 97). The balance of these enzymatic functions determines whether PTM levels reach the necessary threshold to trigger downstream signaling pathways and epigenetic changes.

This regulation affects metabolic adaptation by altering key transcription factors, metabolic enzymes and chromatin configurations. Exceeding the PTM threshold enhances the functionality of genes related to mitochondrial biogenesis, fatty acid oxidation and autophagy, thereby improving metabolic capacity and resilience. Conversely, failing to meet this threshold may lead to inadequate adaptation or metabolic disorders (97, 103).

Both experimental and clinical evidence support the PTM threshold hypothesis. Proteomic analyses show that exercise induces dose-dependent increases in PTMs such as histone lactylation and acetylation, which are associated with improved mitochondrial function and metabolic health indicators. Clinical studies demonstrate that higher-intensity exercise regimens yield more substantial and lasting metabolic benefits, consistent with the notion of a PTM threshold that triggers sustained adaptation (7). The PTM threshold hypothesis proposes that a certain exercise intensity is necessary to induce significant PTM modifications. This hypothesis is based on evidence from metabolic and stress responses, but direct validation in the context of exercise adaptation is still needed. It highlights the importance of achieving a critical level of PTMs to induce lasting metabolic improvements, offering a mechanistic explanation for the dose-dependent effects of exercise and suggesting potential strategies for enhancing metabolic health through PTM modulation.

### 3.3 Interaction model of PTM genetic background and exercise responsiveness

Individual genetic variability strongly influences PTM patterns, directly impacting exercise adaptability and forming a model of the interaction between PTM-related genetic background and exercise responsiveness. Genetic polymorphisms that alter the expression or activity of PTM-related enzymes including acetyltransferases, ubiquitin ligases, deacetylases and lactylation catalysts can cause individual differences in the extent and specificity of exercise-induced PTM modifications, in turn shaping diverse metabolic and physiological responses (109, 112, 113) (Table 2).

**Table 2:** Interaction between PTM genetic background and exercise responsiveness.

PTM-related gene type	Impact of genetic polymorphism	Changes in PTM and exercise responsiveness	References
Acetyltransferase genes (ACLY)	Gain-of-function polymorphism	Increased acetylation level; enhanced mitochondrial biogenesis and metabolic improvement post-endurance exercise (high responder phenotype)	(101, 102)
Ubiquitin ligase genes (Parkin)	Loss-of-function polymorphism	Reduced ubiquitination efficiency; delayed muscle repair and weakened hypertrophy effect after resistance training (low responder phenotype)	(66, 110)
Lactylation-related genes (p300)	Upregulated expression polymorphism	Enhanced lactylation sensitivity; faster activation of metabolic genes and shortened recovery cycle post-HIIT (high adaptation phenotype)	(56, 59)
Deacetylase genes (SIRT3)	Loss-of-function polymorphism	Abnormally elevated acetylation level; accumulation of oxidative stress and delayed fatigue recovery post-exercise (exercise intolerance phenotype)	(98, 100, 101)

This table shows the links between polymorphisms in PTM-related genes, changes in PTM, and exercise response phenotypes. It shows the molecular basis for why different people respond differently to exercise.

This interaction model explains the heterogeneity of exercise outcomes, with some individuals showing substantial metabolic and performance improvements while others display minimal or inconsistent responses. Genetic variants affecting acetylation machinery can modify mitochondrial function and the body's adaptation to energy metabolism. Polymorphisms in ubiquitination pathways may alter protein turnover and autophagy efficiency, potentially influencing post-exercise recovery and cellular remodeling (97, 103). Mass spectrometry now supports scalable “acetylome” and “ubiquitinome” profiling, using anti–acetyl-lysine enrichment and K-ε-GG (diGly) remnant enrichment to map sites at depth (114). Glycoproteomics is also advancing with improved enrichment and MS/MS strategies, making glycosylation omics increasingly feasible (115). Changes in genes regulating lactate metabolism and lactylation enzymes can affect epigenetic responses that govern metabolic memory-related gene expression.

This model clarifies how genetic background interacts with exercise-induced PTM profiles to shape individual responsiveness, integrating genomic data with PTM profiling. Research shows that genetic variants in PTM-related genes are associated with various exercise-induced metabolic adaptations, including mitochondrial biogenesis, fatty acid oxidation and inflammatory regulation (7, 97). Importantly, exercise research already has PTM-omics “platform” datasets that can support machine-learning models. Human training studies have profiled the skeletal muscle acetylome alongside the proteome after HIIT (67). Ubiquitin signaling has also been examined in human skeletal muscle in response to exercise, and current workflows are designed to scale with improved sensitivity (66). By contrast, exercise glycoproteomics is less mature, but the underlying MS technologies are moving quickly and are ready to be applied as datasets grow (115). This interaction model has important implications for personalized exercise prescription. Assessing how an individual's PTM-related genetic background affects exercise efficacy allows the development of tailored training programs matching their unique molecular profile, optimizing health benefits and minimizing adverse effects. It also facilitates pharmacological or nutritional interventions targeting specific PTM pathways to improve exercise responsiveness in genetically predisposed individuals (103).

In summary, the PTM-related genetic background-exercise responsiveness interaction model clarifies the molecular basis of individual differences in exercise adaptation. It highlights the key role of genetic factors in shaping PTM-mediated regulatory networks and lays the foundation for precision exercise medicine, which focuses on improving metabolic health by personalized modulation of PTM dynamics.

## 4 Precise molecular roadmap for exercise prescription

### 4.1 Application of machine learning in exercise PTM fingerprint analysis

Machine learning techniques have shown promise in precision medicine and are increasingly being explored for exercise prescription. While initial results are promising, more research is needed to determine their full potential in developing personalized exercise plans (116, 117). This approach seeks to acquire high-quality multi-omics datasets encompassing proteomics, transcriptomics, metabolomics, and epigenomics, which together illustrate the dynamic landscape of PTMs influenced by different exercise modalities (118). It can also integrate genomics and chromatin-level data (DNA methylation and chromatin accessibility) to connect PTM patterns with upstream regulatory control (119, 120). Advanced data collection techniques like mass spectrometry-based proteomics allow the identification and quantification of various PTMs (phosphorylation, acetylation, ubiquitination, glycosylation) across diverse tissues and temporal contexts (2, 121). Large-scale exercise resources now measure multiple PTM-omes together with other omes across tissues and time, which strengthens model training and validation (119). Recent MS-based PTM resources and models support scalable profiling and prediction across multiple PTM types, including phosphorylation, acetylation, ubiquitination, and glycosylation, which can strengthen exercise PTM model development (69). With these richer PTM layers, multi-omics integration can more clearly capture exercise-specific molecular signatures. Multi-omics integration techniques enhance the clarity and understanding of exercise-specific molecular signatures by amalgamating diverse datasets. Common integration strategies include multiblock correlation methods (DIABLO), matrix-factorization approaches, network/graph-based learning, and deep generative models that can handle missing modalities and batch effects (122). Machine learning algorithms including supervised classifiers such as support vector machines (SVM), random forests, and deep learning models like convolutional neural networks (CNNs) and recurrent neural networks (RNNs) are used to identify PTM patterns linked to different exercise types, intensities, and durations, leveraging a substantial dataset (7, 57). Machine learning models have shown high accuracy in predicting phosphorylation and acetylation sites across various species, highlighting conserved metabolic regulatory mechanisms (123, 124). These algorithms help identify PTM crosstalk, showing how alterations interact to modify protein function and cellular pathways during exercise adaptation (124, 125).

Analyses using machine learning have led to the development of extensive databases connecting exercise, PTMs, and disease. These databases combine molecular signatures with clinical phenotypes, enabling the identification of PTM biomarkers that predict individual exercise responsiveness and disease risk (126, 127). Such databases are highly valuable for precision exercise medicine, as they allow stratification of individuals based on molecular response profiles and tailoring of exercise prescriptions to each group. When multi-omic layers are profiled in the same samples, integrative models can also prioritize candidate regulators (transcription factors, kinases, acetyltransferases, and E3 ligases) that may coordinate PTM programs across tissues (128). Recent exercise-related multi-omics studies illustrate this by integrating methylome–proteome or methylome–transcriptome layers to link CpG changes with downstream protein or RNA programs (including BETA-based integration and cistrome-informed regulator prediction) (129). Furthermore, applying natural language processing and text mining techniques to biomedical literature accelerates the acquisition of PTM-related information, thereby improving the quality of training datasets and the efficacy of machine learning models (130). The use of machine learning in exercise PTM fingerprint analysis provides a robust framework for understanding the molecular language of exercise, paving the way for personalized and mechanistically informed exercise prescriptions.

### 4.2 Personalized exercise program design based on PTM biomarkers

Personalized exercise prescriptions based on PTM biomarker profiles represent a transformative approach to enhancing interventions for metabolic, neurodegenerative, and oncological diseases. Importantly, the same PTM-omics framework could be deployed proactively before structured exercise training begins. Baseline PTM fingerprints collected at rest may stratify individuals with and without clinical risk factors along a health risk continuum. This may help map early molecular risk states, guide safer starting loads, and support prevention-oriented, personalized exercise prescriptions before overt disease develops. Emerging PTMs linked to metabolic stress and aging biology, such as glycation and lactylation, further support the plausibility of pre-training PTM-based risk mapping (131, 132). This approach requires well-powered longitudinal cohorts that include people with low and elevated baseline risk. It also requires repeated PTM-omics measurements and careful control of major confounders, including diet, medication, circadian timing, and training status. These steps are needed to distinguish stable risk signatures from transient state effects. Baseline circulating proteomic signatures combined with machine learning can predict individualized metabolic responsiveness to exercise training in men with prediabetes (133). Large prospective plasma proteomics studies also show that circulating molecular signatures can predict incident diseases years before diagnosis. Together, these findings support the feasibility of pre-training molecular risk mapping (134–136). In parallel, deep learning approaches can help identify disease-related PTM signatures and support risk stratification (137). However, this hypothesis still needs longitudinal validation. Future studies should test whether PTM-resolved features add predictive value beyond standard clinical risk scores and whether training shifts these signatures toward a healthier profile (138).

Building on this proactive risk-stratification concept, accumulating evidence across major disease domains suggests that PTM-informed readouts can further refine exercise prescription when specific pathophysiological contexts are considered. In metabolic disorders like diabetes and hypertension, PTMs affect important signaling pathways that control insulin sensitivity, lipid metabolism, and how the body reacts to inflammation. Exercise-induced changes in proteins related to mitochondrial function and glucose metabolism are associated with improved metabolic flexibility and glycemic control (139, 140). Tailoring exercise plans according to PTM signatures enables precise adjustments to the intensity, duration, and type of exercise, optimizing outcomes while minimizing adverse effects. Neurodegenerative diseases are characterized by abnormal PTMs that result in protein misfolding and aggregation, as observed in Alzheimer's and Parkinson's diseases. Exercise interventions influence PTMs such as phosphorylation and acetylation on neuroprotective proteins, enhancing synaptic plasticity and reducing neuroinflammation (141, 142). Personalized exercise programs that incorporate PTM biomarkers can effectively target specific molecular deficiencies, enhancing neuroprotection and promoting functional recovery.

**Figure 4:**
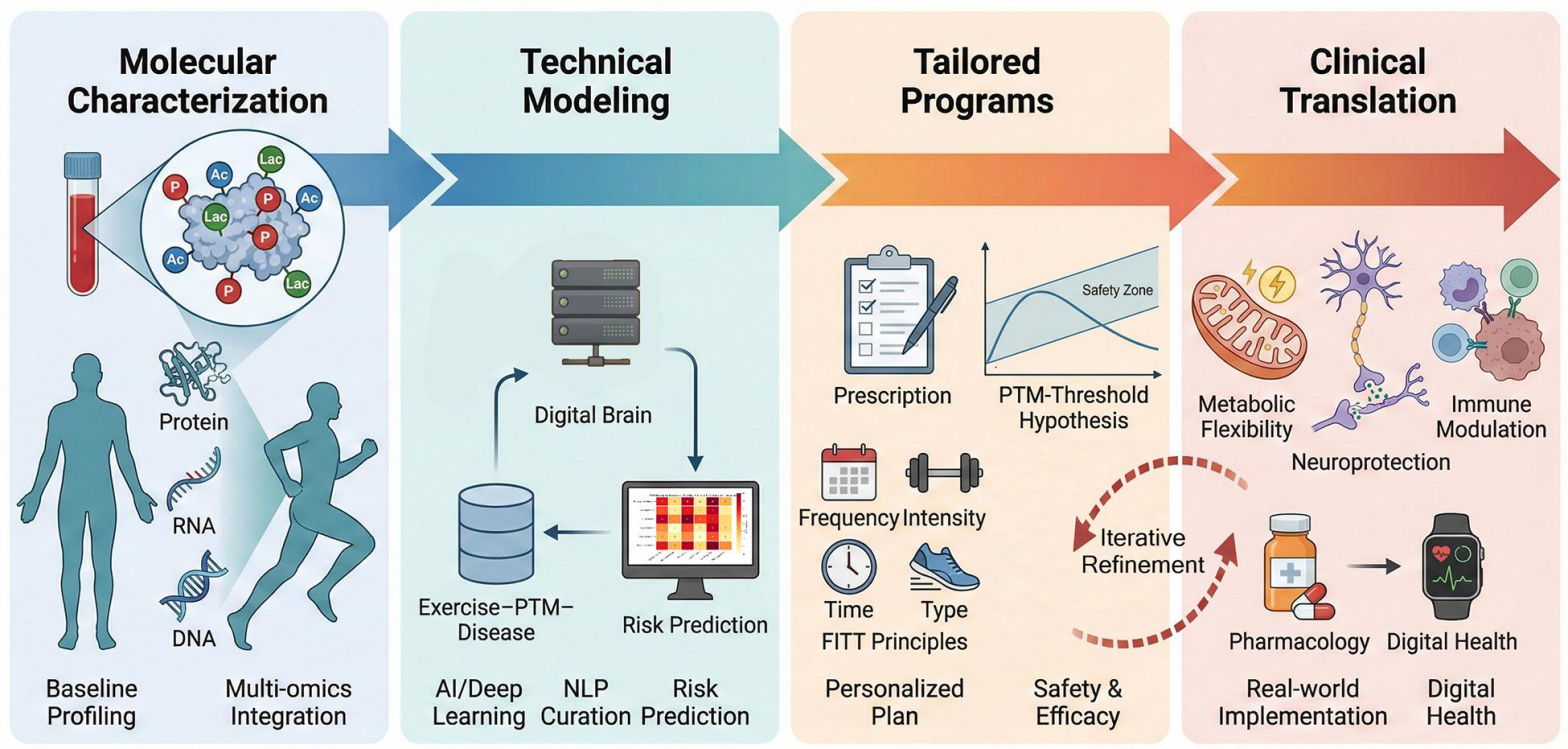
Implementation roadmap of precision exercise prescription driven by PTM signatures and emerging technologies.

In oncology, exercise prescription based on PTM profiles shows promise for reducing treatment-related toxicities and enhancing survival outcomes. Research has identified exercise-responsive PTM biomarkers associated with immune modulation and tumor microenvironment remodeling, enabling the development of customized rehabilitation protocols (143, 144). PTM-based stratification optimizes exercise timing and intensity, enhancing cardiovascular protection and functional capacity in cancer patients (145, 146). The utilization of PTM biomarkers in clinical decision-making requires robust validation via multi-omics integration and extended monitoring. This ensures that exercise prescriptions are consistently modified to accommodate evolving molecular landscapes. This biomarker-driven methodology demonstrates effectiveness across diverse demographics, including elderly individuals, neurological patients, and cancer survivors, underscoring its broad applicability (147, 148). Personalized exercise programs informed by PTM biomarkers represent a precise, mechanism-based approach to enhancing outcomes for patients with various chronic diseases (Figure 4).

Figure 4 shows a four-stage roadmap for precision exercise prescription. First is molecular characterization, which integrates baseline PTM profiling at rest for molecular risk stratification with multi-omics (proteomics, transcriptomics and related layers) to capture dynamic exercise-responsive PTM patterns, including phosphorylation and acetylation. Second is technical modeling, which applies AI, machine learning (supervised models and deep learning) and NLP to integrate these datasets, predict baseline risk and PTM responses, and curate an “exercise–PTM–disease” association database. Third is tailored programs, which design and iteratively refine personalized exercise plans based on PTM biomarkers, genetic background and the PTM-threshold hypothesis to optimize safety and efficacy across disease contexts. Fourth is clinical translation, which combines PTM-targeted pharmacology with digital health and tele-rehabilitation tools to support real-world implementation from metabolic management to oncology recovery. This roadmap links molecular PTM signatures to actionable precision interventions.

### 4.3 New targets in exercise pharmacology and clinical translation

Recent elucidation of PTM-regulated molecular pathways underlying exercise-derived benefits has identified emerging pharmacological targets, which mimic or amplify exercise-induced metabolic and neuroprotective adaptations. This advancement positions exercise pharmacology as a viable supplementary therapeutic strategy (149–151). Specifically, this discipline centers on developing molecular agents that modulate PTM pathways, with the goal of replicating exercise's metabolic homeostasis and neuroprotective outcomes (152, 153). Notably, small molecules targeting lactylation pathways such as histone H3K18 lactylation modulators have shown promise in promoting M2 macrophage polarization and alleviating atherosclerosis, paralleling the immunometabolic reprogramming triggered by moderate-intensity aerobic exercise (154). Additionally, lactate receptor agonists are under investigation for enhancing post-stroke neuroregeneration, leveraging lactate-mediated signaling pathways activated during sustained physical activity (155, 156). For rare conditions like glycogen storage disorders, PTM profile-guided exercise rehabilitation strategies are being customized into individualized interventions, specifically enhancing muscle energy metabolism and functional recovery (157).

Translating these insights to clinical practice requires thorough evaluation of candidate molecules, assessing safety, efficacy, and pharmacodynamic properties, coupled with biomarker-driven patient stratification to boost therapeutic responsiveness. Advancements in precision medicine frameworks, which leverage genomic, epigenomic, and proteomic data, support the development of combinatorial regimens. These regimens integrate personalized exercise prescriptions with pharmacological modulation of PTMs (158, 159). The adoption of digital health technologies and tele-rehabilitation platforms improves treatment accessibility and adherence, enabling real-time tracking of molecular and functional therapeutic responses (160, 161). Ultimately, integrating PTM biology, exercise science, and pharmacology advances the development of targeted interventions. These interventions harness exercise's molecular mechanisms. This provides emerging therapeutic options for diverse patient groups and expedites the clinical translation of exercise mimetics (2, 162, 163).

## 5 Conclusions and perspectives

The study of exercise-specific PTM expression profiles represents a notable leap in understanding the molecular mechanisms governing physical activity. This distinctive framework for classifying exercises deepens insights into the distinct molecular responses triggered by aerobic, resistance, and high-intensity interval training. It provides a more detailed and mechanistic perspective for exercise physiology research. From a professional standpoint, this transition not only strengthens the theoretical foundation of exercise physiology. It also addresses key deficiencies in translating molecular biology findings into actionable clinical exercise guidelines.

Notably, the elucidation of PTM decoding mechanisms underlying metabolic memory, alongside the proposed “PTM threshold” hypothesis, drives this progress. These concepts establish a robust molecular basis for linking exercise intensity to metabolic adaptation. They offer a measurable approach to understanding how different exercise loads yield long-term health benefits. Current research indicates that traditional exercise intensity measures have proven effective in population-level studies. The integration of PTM dynamics, however, adds a more personalized and mechanistic dimension aligned with modern precision medicine goals.

Equally important, applying machine learning to analyze PTM fingerprint profiles marks another key advancement. This method simplifies the interpretation of complex PTM datasets. It enhances the development of precise exercise prescriptions tailored to individual molecular signatures. The clinical implications are meaningful, as this methodology shows potential for improving rehabilitation protocols. It addresses metabolic disorders and may enhance outcomes in rare diseases with variable exercise responsiveness. Thorough assessment of these findings is essential, considering methodological diversity and machine learning limitations such as training dataset biases. This ensures clinical application is grounded in robust validation.

Theoretical advancements in this field have laid a molecular foundation for resolving inter-individual variability in exercise responses. These progressions provide mechanistic insights that advance the emerging field of exercise pharmacology. They also create new opportunities for therapeutic interventions in rare disease rehabilitation (164). The complexity of PTM regulation and its crosstalk with other molecular networks demands careful analysis of current data. It requires acknowledging both the potential and uncertainties in this rapidly evolving research area (165).

Future research should prioritize integrating multi-omics datasets to comprehensively validate PTM regulatory mechanisms. Integrative approaches will significantly deepen understanding of the “PTM threshold” hypothesis. They will also clarify the molecular changes induced by exercise (166–168). Furthermore, the advancement of personalized exercise medicine will depend on rigorous longitudinal studies and clinical trials. These studies convert molecular findings into effective, tailored interventions. Through interdisciplinary collaboration and integration of emerging technologies, the discipline is set to transform exercise prescription and application for health maintenance and disease management (169–171). Beyond optimizing prescriptions for established disease, a key next step is to build population-scale baseline PTM atlases across the health-to-disease continuum and link them to incident clinical outcomes, enabling discovery of PTM-based risk signatures prior to training. Translationally, this would support a prevention-first model in which baseline PTM profiles inform early, individualized exercise prescriptions and monitoring strategies, while acknowledging that clinical deployment will depend on prospective validation and standardized pre-analytics.

The development of exercise-associated PTM patterns and their corresponding analytical frameworks constitutes a impactful transformation with far-reaching implications. This body of work synthesizes diverse research perspectives and findings. It advances the molecular understanding of exercise and facilitates the development of precision exercise medicine. Continued validation and expansion of these findings are essential. They will fully realize the clinical potential of these insights and enable tailored exercise interventions that optimize health outcomes across diverse populations.
